# Implementation of gestational weight gain guidelines - what’s more effective for ensuring weight recording in pregnancy?

**DOI:** 10.1186/s12884-018-2162-x

**Published:** 2019-02-12

**Authors:** Shelley Wilkinson, Michael Beckmann, Elin Donaldson, Sally McCray

**Affiliations:** 1 Department of Dietetics and Foodservices, Mater Health, Brisbane, Queensland 4101 Australia; 2 Mater Research Institute – University of Queensland, Mothers, Babies and Women’s Theme, Brisbane, 4101 Australia; 3 Mater Mothers’ Hospitals, Mater Health, Brisbane, 4101 Australia

**Keywords:** Antenatal, Weight recording, Guidelines, Implementation, Translation

## Abstract

**Background:**

Pregnant women who gain weight in accordance with guidelines have the lowest risk of pregnancy and birth-related complications. However, evidence-practice gaps often exist. To address pregnancy weight management barriers, a stepped implementation science approach was used, comprising targeted in-services, provision of scales for clinic rooms, and changes to routine weight recording in a hospital electronic medical record. The aim of this study was to assess the cumulative influence of evidence-based interventions on staff’s compliance to recording of antenatal weights.

**Methods:**

Retrospective data analysis of weight recording over three 15-month cohorts across April 2014–December 2017. Variables calculated from data included: proportion of women with weight recorded at booking and proportion of women who had a weight recorded at each visit. Generalised estimating equation modelling was used to examine differences in weight recording compliance rates between cohorts, pre-pregnancy body mass index categories, model of care and clinicians.

**Results:**

There were approximately 13,000 pregnancies in each cohort. The proportion of women who had a weight recorded at each visit per cohort differed significantly between cohorts from 4.2% (baseline), 18.9% (scales and in-services) to 61.8% (medical record prompts), *p* < 0.001.

**Conclusion:**

Significant improvements were achieved through systematic barrier analysis and subsequent mapping and implementation of appropriate and effective interventions. Improvements were observed across the entire service, in all models of care with all professional groups demonstrating increased recording of weights.

## Introduction

Pregnant women who gain weight in accordance with the US Institute of Medicine (IOM) guidelines, also adopted for use in Australia [1–4], have the lowest risk of pregnancy and birth-related complications. Their infants are also at reduced risk of incurring a chronic disease during their adult lives [1]. However, existing service processes and delivery of care does not always support women to adhere to best practice guidelines and achieve these outcomes. Even when good evidence is available to support maternal behavioural change, health professionals do not necessarily adopt it, or disseminate it to women [5–8]. This is a broader problem in healthcare delivery, also recognised in many acute care settings [9–11].

A recent systematic review and meta-analysis noted that weighing as a stand-alone intervention does not reduce excessive gestational weight gain (GWG) [12]. However, it has been clearly demonstrated that interventions that include dietary advice, and physical activity, supported by ongoing weight monitoring, can prevent excessive GWG and result in more women across all body mass index (BMI) categories achieving weight gain within the correct ranges [13]. This approach has been shown to be highly efficacious when delivered by obstetricians and midwives and supported by dietitians, in an antenatal setting [14]. Further, many clinical guidelines (e.g. IOM, Queensland Health [1, 15]) rely on accurate weight records for timely clinical interventions, such as referral to anaesthetists and dietitians and for undergoing glucose tolerance testing.

Simply producing and disseminating guidelines does not always effect practice change. Evidence-practice gaps may continue to exist [16] and to close this gap a systematic and theory-driven process (i.e. the implementation science methodology) is essential. This requires an assessment of influencing factors (barriers/enablers) and an implementation and evaluation plan that uses strategies to overcome and address barriers [17]. The use of a framework to underpin the process is highly recommended [18].

To address the management of the GWG evidence-practice gap at our tertiary maternity service we took a system-wide approach, using the theoretical domains framework (TDF) [17] to classify barriers, and used the behaviour change wheel (BCW) [19], articulating with the TDF, to design effective interventions. Barriers to guideline adherence were informed by serial surveys in 2011 [5] and 2014 [6] and included gaps in GWG knowledge (goals per BMI category, BMI cut-offs), inconsistent weighing and monitoring and recording practices, and staff confidence in their own capabilities to counsel women who were overweight, obese, and/or experiencing excessive GWG. These barriers were classified in the TDF domains of: Knowledge, Skills, Social and Professional Role/Identity, Beliefs about capabilities, and Environmental context and resources [5].

Interventions introduced following the 2011 survey to overcome the identified barriers were delivered within existing resources and aimed to increase capacity and support within clinic. This was operationalised as increased dietetic time in the antenatal clinic comprising group and individual appointments, and delivery and follow up of the ‘Healthy Start to Pregnancy’ initiative (an evidence-based behaviour change workshop) [20] (to address the barrier of Environmental context and resources and to address the extra support required beyond staff’s perceived capabilities). In addition, all women were given a ‘Mater Personalised Pregnancy Weight Tracker’ (‘Weight Tracker’) developed in-house by the maternity hospital dietitians [21] with associated staff training in its use around GWG. This addressed the barriers of Knowledge, Skills and staff’s confidence in their capabilities.

Following the 2014 survey, compliance with individual elements of the guideline recommendations improved, such as improved (accurate) documentation of pre-pregnancy BMI and discussing and correctly advising women about GWG goals, however overall guideline adherence did not improve [6]. Subsequent interventions related to identified gaps were implemented in stages including:

- Obtaining scales for each antenatal clinic room (2014),
- staff in-services training outlining and incorporating the evidence in to practice via voice over PowerPoint presentation (2014), and.
- removal of default ‘skip’ option from the electronic record weight recording to promote mandatory recording (September 2016).

These addressed the TDF barrier domains of knowledge (through enablement/modelling), skills (through training), professional and social role/identify (through education/environmental restructuring) and environmental context and resources (through environmental restructuring).

The aim of this study was to assess the cumulative influence of these interventions on staff’s compliance to recording of antenatal weights.

## Methods

### Design and participants

This retrospective study analysed data recorded by midwifery and obstetric clinicians from all models of care within the publically-funded Mater Mothers Hospital (MMH) antenatal service, across three cohorts over three periods of 15 months. Cohort 1 measured baseline weighing practices (April 2014–June 2015), cohort 2 measured effect of the availability of scales on weighing practices (July2015-September2016), and cohort 3 measured effect of mandatory weight recording in Matrix (the hospital’s electronic maternity data set: Meridian Health Informatics) on weighing practices (September 2016–December 2017). The models of care include the general and specialist antenatal clinics (e.g. pregnancy after loss; high risk; young women’s clinics, indigenous clinics, refugee clinics; general practitioner clinics) as well as midwifery group practice and community midwifery clinics.

### Procedure and data analysis

Data were extracted from Matrix in three 15 month time periods. The variables of interest were: 1. Visit data (date of first visit; number of antenatal visits; whether weight was recorded at each visit; clinician type who recorded weight at visit; model of care (MOC) for each visit) and 2. anthropometric data (weight at booking; height; pre-pregnancy body mass index (ppBMI). Overall GWG was not able to be analysed due to the low numbers of recorded weight in cohorts 1 and 2.

Women’s characteristics were summarised using frequencies and percentages for categorical variables and means (standard deviation (SD)) or medians (interquartile range (IQR)) for continuous variables. Associations between cohort and categorical variables were examined using a Pearson Chi-squared test. Differences in mean heights and median weight measurements were examined by ANOVA and Kruskal -Wallis tests, respectively. Generalised estimating equation logistic model was used to examine differences in weight recording compliance rates between cohorts by patient pre-pregnancy BMI categories, models of care or clinicians. The exchangeable working correlation structure and robust variance estimation were used to account for correlation between multiple visits within a patient. The level of statistical significance was set at 0.05. Statistical analysis was performed using STATA 15.1.

### Ethical considerations

Ethical approval was obtained from the hospital’s Human Research Ethics Committee (HREC/14/MHS/119. Amendment AM01).

## Results

There were approximately 13,000 pregnancies in each cohort (Table 1). Women’s median pre-pregnancy weight was similar across each cohort (*p* = 0.10). The remaining anthropometric measures (height, median ppBMI, booking weight) and gestation weeks at booking were all statistically (but not clinically) significantly different (all *p* < 0.001).

**Table 1:** Women’s anthropometry and pregnancy details across the 3 study cohorts

	Cohort 1 (n women = 13,577)	Cohort 2 (*n* = 13,481)	Cohort 3 (*n* = 12,861)	*p*-value
Date range (months)	April 2014–June 2015 (15)	July 2015–September 2016 (15)	October 2016–December 2017 (15)	
pp weight (kg), median (IQR)	63.0 (56.0–72.0)	63.0 (55.0–73.0)	63.0 (55.0–73.0)	0.10
Weight at booking (kg), median (IQR)	70.0 (62.0–80.0)	68.0 (60.0–79.0)	69.0 (61.0–79.3)	< 0.001
ppBMI, median (IQR)	22.7 (20.4–25.9)	22.9 (20.5–26.5)	23.1 (20.7–26.8)	< 0.001
Gestation at booking (weeks), median (IQR)	21.5 (15.2–29.4)	18.3 (14.6–21.3)	20.0 (14.6–22.6)	< 0.001

Data are presented as % (n/N) for categorical variables. *IQR* interquartile range, *pp* pre-pregnancy, *BMI* body mass index

Consistency of recording of booking weights differed significantly across cohorts (*p* < 0.001) (Table 2). The proportion of women who had a weight recorded at each visit per cohort (overall) also differed significantly between cohorts 1, 2, and 3 from 4.2 to 18.9% to 61.8%, *p* < 0.001 (Table 2). This had a similar pattern across clinician groups, MOC, and ppBMI categories (all *p* < 0.001). The compliance rates for weight recording (overall) in cohort 2 and cohort 3 were 4.54 (95% CI 4.24–4.86) times and 15.0 (95% CI 14.2–16.0) times as high as the compliance rate in cohort 1, respectively. Overall, women with ppBMI categories < 18.5 kg/m^2^ and > 30 kg/m^2^ were more likely to have their weights at subsequent visits recorded (RR 1.10, 95% CI 1.03–1.18 and RR 1.11, 95% CI 1.06–1.17, respectively) compared to those with a ppBMI 18.5–24.9 kg/m^2^ (healthy BMI range).

**Table 2:** Service use and weight recording practices across the 3 study cohorts

	Cohort 1 (*n* visits = 38,785)	Cohort 2 (*n* = 32,694)	Cohort 3 (*n* = 24,623)	*p*-value
Date range (months)	April 2014–June 2015 (15)	July 2015–September 2016 (15)	October 2016–December 2017 (15)	
Proportion of women with weight each visit recorded (overall) (%)	4.2%	18.9%	61.8%	< 0.001
Weight recorded at booking visit	92.2%	84.8%	92.2%	< 0.001
Proportion of women with weight each visit recorded (%) – By clinician group				< 0.001
by Midwives	4.0%	20.7%	62.5%	
by Doctors	4.1%	7.6%	53.3%	
By model of care				< 0.001
In the hospital	4.0%	15.7%	56.6%	< 0.001
In the community	4.0%	24.7%	69.8%	< 0.001
By ppBMI category				< 0.001
< 18.5 kg/m^2^	5.7%	22.0%	64.6%	< 0.001
18.5–24.9 kg/m^2^	3.8%	17.3%	60.9%	< 0.001
25–29.9 kg/m^2^	3.6%	20.0%	62.0%	< 0.001
30 + kg/m^2^	5.8%	21.5%	63.5%	< 0.001

Data are presented as % (n/N) for categorical variables. *IQR* interquartile range, *pp* pre-pregnancy, *BMI* body mass index

## Discussion

This study of consistency of routinely collected weight recordings clearly demonstrates the cumulative influence of targeted interventions on staff’s adherence to recording of antenatal weights. These significant improvements were achieved through systematic barrier analysis and subsequent mapping and implementation of appropriate and effective interventions [17, 19]. Improvements were observed across the entire service, in all MOC, with all professional groups demonstrating increased recording of weights. The cumulative changes positively influenced the recording of weight during women’s pregnancies. More weights were recorded at a visit in each cohort for women in the underweight and obese BMI categories.

Under-recording of weights is a well-recognised issue in the acute healthcare setting despite its acknowledged importance on patient and clinical outcomes [10]. The low rate of weights recorded is similar to that seen in acute care hospital settings, with audits documenting 2, 18, 22 and 28% of weights being recorded [10, 11], despite 80% of staff acknowledging its importance on patient/clinical outcomes [10]. Another Australian/New Zealand audit of nutrition care practices in hospital wards revealed only 32% of 370 wards surveyed documented weights, with 55% only recording it “when requested” [9]. In the broader health care arena, correct and timely documentation of weight is important for safe prescribing practices, manual handling, skin integrity management, and identifying nutritional risk [10]. The importance of clearly documented weights and therefore rate of GWG is similarly important in maternity care, informing anaesthetic care-planning, allowing detection of potential metabolic complications (GDM), determining resource requirements (beds, blood pressure cuffs, ultrasound processes), and as an indicator of nutritional risk.

Fealy et al’s systematic review and meta-analysis did not find routine weighing reduced excessive GWG [12]. However, the two included studies did not incorporate any dietary, physical activity, or behavioural change interventions known and shown to be required for effective healthy weight management during pregnancy [13, 22]. The authors opined that routine weighing is as important as blood pressure measurement [12]. Routine weighing is acceptable to women and has not been shown to increase anxiety or distress [23]. It is also a valuable opportunity to act and counsel women to support a healthy pregnancy and can assist clinicians providing appropriate and timely interventions [24].

Despite the absence of data to compare GWG across the three cohorts, we are able to draw on self-reported data from a smaller population from the same study period to provide a proxy measure of the interventions’ effectiveness [25]. In this paper examining women’s service engagement, health behaviours and GWG across 2014–17 (n421) with a cohort of women surveyed at the opening of the MMH in 2008 (n102) we were able to demonstrate that the proportion of women experiencing correct GWG increased from 23.2 to 38.6% (with excessive GWG decreasing from 57.3 to 32.6%), *p* < 0.001 [25]. We noted the significant increase in women gaining weight within the recommended IOM guideline ranges was a very promising outcome which was lower than many recent studies that used similar methodologies (E.g. 38% [26], 39% [27], 42% [28], 47% [4], and 52% [29]). Furthermore, we demonstrated that a greater proportion of women accessed the nutrition services in 2014–17 compared with 2008 (19.7% vs 9.9%) and rated nutrition resources provided and/or viewed favourably (> 3.5 out of 5) [25].

Simple system changes appear to be most effective for sustainable outcomes in this instance of antenatal weight recording. It has been noted that reliance on memory less consistently elicits behaviour change compared to routine structural changes [30]. Thus, when scales were available, the removal of a default skip in the weight recording process became an ‘electronic reminder’. These interventions elicited greater improvements than have previously been documented for reminder systems (usually only ~ 4%) [31], perhaps through being uniquely tailored to the local barriers in place. A recent review of eHealth technologies in hospital practice presented strong evidence that electronic medical records can substantially increase clinician adherence to guidelines and accuracy and completeness of clinical information, especially when used at the point of care and integrated into workflows [32].

This study’s strengths were its large sample size, extended period of measurement, and the use of the implementation science approach to devise effective strategies to overcome known barriers to the practice of consistent regular weighing of women. Limitations relate to the inability to determine the effect of the increased weight recording on GWG and the resultant effects on clinical outcomes such as shoulder dystocia, induction of labour, operative delivery, and neonatal hypoglycaemia. Furthermore, due to the relatively late timing of the women’s booking visit there is the potential for inaccuracies of pre-pregnancy weight reporting. However, it has been demonstrated that there is a high correlation between measured and self-report anthropometry [33]. There is also the potential for an unhealthy weight gain trajectory to be established with later bookings which should be addressed in future interventions.

Additionally, we do not know how the experience of weighing affected women, or the clinicians delivering care, and if this impacted referral practices. However, the serial improvements reflect that the practice of weight recording increased across all models of care which is likely to have had positive effect on women’s care overall. Other studies have documented that women like and expect to be weighed if it is performed in a respectful manner [23, 34]. Although some staff report feeling anxious about weighing women [35–37] we are providing additional training and support to help them overcome this barrier. This may also address the less than 100% compliance rate for weight recording.

To enable monitoring of practices against guidelines, including rate of and overall GWG, we will investigate the possibility of modifying the dataset to easily capture (and extract) weight at K28 and K36. This will also allow linking routine monitoring of GWG to pregnancy outcomes. We will also investigate the possibility of incorporating the paper Mater Personalised Pregnancy Weight Tracker [21] into the electronic record. This operationalizes a very effective behaviour change intervention, audit and feedback [38, 39], by providing real-time data on a woman’s progress and prompting staff on the required actions for when a woman deviates from her recommended weight trajectory.

## Conclusion

In our recent paper presenting service-wide changes to support healthy GWG, we noted that the effectiveness of changes can be difficult to tease apart when a suite of changes are implemented concurrently [6]. Using a stepped approach we have been able to demonstrate the cumulative effectiveness of evidence-based interventions targeted to known barriers [38] and have reinforced the knowledge that interventions based on environmental restructuring, restriction (of processes or practices), enablement, incentivisation and/or coercion can be more effective than relying on an individual’s choice to facilitate the “automatic” delivery of best practice [21].

## Abbreviations

ANOVA: Analysis of variance
BCW: Behaviour change wheel
BMI: Body mass index
GDM: Gestational diabetes mellitus
GWG: Gestational weight gain
HREC: Human research ethics committee
IOM: Institute of Medicine
IQR: Interquartile range
MMH: Mater Mothers Hospital
MOC: Model of care
ppBMI: Pre-pregnancy BMI
SD: Standard deviation
TDF: Theoretical domains framework
